# Decision of the Supreme Court of the State of New York on the Question of the Validity of the Charter of the College of Physicians and Surgeons

**Published:** 1883-03

**Authors:** 


### Decision of the Supreme Court of the State of New York on the Question of the Validity of the Charter of the College of Physicians and Surgeons.

*The People of the State of New York, Respondents, vs. George W. Cothran and Others, Appellants.*

Medical College—It cannot be formed under Chapter 319 of 1848, as explained by Chapter 51 of 1870—What educational institutions may be formed under these acts—Chapter 319 of 1848, providing for the incorporation of benevolent, charitable, scientific and missionary societies, as explained by the declaratory act of 1870 (Chap. 51), does not provide for or authorize the incorporation of medical colleges, to give instruction in the art of medicine and surgery for a compensation to be secured by them, and to issue and grant diplomas to graduates—Only such educational institutions can be formed under the said acts as are connected with or controlled by some Christian Church or association.

Appeal from a judgment entered upon an order of the Erie Special Term, overruling a demurrer to the complaint.

Tracy C. Becker and George Clinton for the appellants.

Hamilton Ward, Attorney-General, and A. G. Rice, for the respondents.

SMITH, P. J.:

The defendants are charged in this action with acts of usurpation, in assuming to be a body corporate, without due authority of law, and the relief asked for is that they be adjudged not to be legally incorporated, and that they be restrained from acting as a corporation. The complaint alleges that in March, 1879, the defendants assumed to be incorporated as a medical college in the city of Buffalo, under the provisions of Chapter 319 of the Laws of 1848, and of the several acts amending the same, and as such corporation to give instruction in the art of medicine and surgery, for which they demanded and received compensation, and to issue and grant diplomas to graduates, and to do all other acts pertaining to and ordinarily done by incorporated medical colleges.

The question is whether a medical college can be lawfully incorporated under the acts referred to.

The act of 1848 is entitled, "An act for the Incorporation of Benevolent, Charitable, Scientific and Missionary Societies;" and the first section of the act authorizes any five or more persons possessing the qualifications prescribed by the act to associate themselves for "benevolent, charitable, scientific or missionary purposes." If that act stood alone, a question might well be raised as to whether it was intended to authorize the formation of societies for the purpose of carrying on medical or other colleges, or any institution whatever, which is primarily and exclusively "educational," and especially one in which a compensation is demanded for the instruction furnished. An institution of the latter kind could hardly be regarded as a "benevolent," "charitable" or "missionary" institution, within the ordinary meaning of those terms. In a certain sense a medical college may be termed a "scientific" institution, as it is designed to give instruction in the science of medicine, but the idea most readily suggested by the use of the term "scientific" in the act referred to, is that the legislature intended merely to authorize individuals to associate themselves together for the purpose of mutual co-operation in scientific investigation and pursuits.

The legislature has solved the doubt, to a certain extent, by a subsequent act declaratory of the meaning of the act of 1848. We refer to Chapter 51 of the Laws of 1870. Its first section declares that the act of 1848 shall be deemed to authorize the incorporation of any society for the purpose of establishing and maintaining "any educational institution, or chapel, or place of Christian work, or any parsonage, rectory or official residence of any bishop, pastor or minister of any Christian Church or association." It is to be observed that the act of 1870 does not add to or vary the words of the act of

1848, although in its title it purports to "amend" it; but it simply declares the meaning of the earlier act, and it declares that certain educational institutions are within its scope. The defendants contend that it authorizes the establishment and maintenance of any educational institution, while it is insisted on the part of the plaintiff that it is limited to such educational institutions as are connected with or controlled by some Christian Church or association. We incline to the latter interpretation for several reasons:

*First.* It is a well-settled rule of the common law that all legislative grants of privilege are to be liberally interpreted in favor of the public, and as against the grantees of the monopoly, franchise or charter, to be strictly interpreted. Whatever is not unequivocally granted in such acts is taken to have been withheld. (Sedg. on Const. and Stat. Law, 338, 339, and cases there cited in note.) That rule is fully applicable to a statute authorizing the formation of an unlimited number of corporations, and especially corporations vested with powers so directly and vitally affecting the interests of the public as the licensing of persons to practice the art of medicine.

*Second.* The maxim *noscitur a sociis* applies. The term "educational institution" is classed in the statute with the terms "chapel," "place of Christian worship," and "parsonage, rectory or official residence of any bishop, pastor or minister of any Christian Church or association," all of which imply Church control.

*Third.* The uniform policy of the State, as evinced by its legislation (unless the acts of 1848 and 1870 are exceptions), has been to create medical colleges by special charter or by special act of the Board of Regents (Laws 1853, Chap. 184) prescribing various requisites, conditions and limitations looking to the increased usefulness of the institutions and to the public good. An intention to depart from that policy should not be implied from words of doubtful signification.

*Fourth.* Furthermore, the grant of a franchise so important to the grantees and to the public as that of forming a corporation for the establishment and maintenance of a medical college with the powers claimed by the defendants ought not to be spelled out by judicial construction of doubtful language. The presumption is that if the legislature intended to confer it they would have done so in clear and unmistakable language.

The third and fourth sections of the act of 1870, relied upon by the defendants, do not strengthen the construction which they contend for. There is nothing in those sections to indicate that they refer to any other universities or colleges than those which are under Church control.

The circumstance that the Regents of the university, in their reports, and other State officers, in their official utterances, have, from time to time, recognized the existence of medical colleges assuming to have been formed under the acts of 1848 and 1870 has but little bearing upon the question as to whether such institutions were legally created. The most that can be said is that those officers, finding them in existence, treated them as *de facto* corporations, without assuming to pass upon the question of the validity of their organization, a matter beyond the scope of their official authority.

We think the judgment and order of the Special Term should be affirmed.

Hardin and Haight, JJ., concurred.

Judgment and order affirmed, with costs.

